# Polypharmacy and Its Spatial Clustering in Japan: An Ecological Study of the Standardized Polypharmacy Ratio

**DOI:** 10.7759/cureus.91228

**Published:** 2025-08-29

**Authors:** Shohei Ono, Yusuke Iizuka, Shinshu Katayama

**Affiliations:** 1 Anesthesiology and Critical Care Medicine, Jichi Medical University Saitama Medical Center, Saitama, JPN; 2 Emergency and Intensive Care Medicine, Tokyo Metropolitan Tama Medical Center, Tokyo, JPN; 3 Anesthesiology and Intensive Care Medicine, Jichi Medical University, Tochigi, JPN

**Keywords:** ecological research, information literacy, moran’s i, ndb open data, polypharmacy, spatial autocorrelation

## Abstract

Background: Polypharmacy, defined as the concurrent use of multiple medications, poses significant health risks, particularly among aging populations. While polypharmacy is a recognized concern, limited research has examined its spatial distribution or its association with demographic and socioeconomic factors. This study aimed to examine the spatial patterns of polypharmacy across Japan and identify regional characteristics associated with higher polypharmacy rates. This study could contribute to evaluating the effectiveness of current local and national policies and may also inform future policy initiatives.

Methods: An ecological study was conducted across 335 local health units in Japan using data from national health, demographic, and geographic databases. Polypharmacy was defined as prescriptions containing seven or more drugs, and the standardized polypharmacy ratio (SPR) was calculated by age-group population. Spatial autocorrelation of SPR was assessed using Moran’s I statistic. Clustering analysis incorporating SPR and regional variables identified distinct high-risk areas.

Results: The prediction model for polypharmacy prescriptions achieved an R² of 0.98, indicating high accuracy, though SPR remained heterogeneous. Significant spatial autocorrelation was observed for both polypharmacy prescriptions (Moran’s I = 0.4; P < 0.001) and SPR (Moran’s I = 0.24; P < 0.001), highlighting regional clustering. Clustering analysis identified four groups by polypharmacy risk (critical, high, moderate, and low). High-SPR areas were associated with higher population density, a greater proportion of younger adults (ages 20 to 60), and increased levels of education, income, and tertiary industry workers.

Conclusion: Polypharmacy in Japan exhibits significant spatial clustering, with higher rates in urbanized regions driven by demographic and socioeconomic factors. Region-specific interventions addressing these unique characteristics are essential for improving polypharmacy management.

## Introduction

Polypharmacy, defined as the concurrent use of multiple medications, commonly five or more, has become a pressing public health issue, though definitions may vary [1]. Previous studies have demonstrated its potential to induce adverse effects, including falls, cognitive decline, and malnutrition conditions that contribute to overall health deterioration [2,3]. Such adverse outcomes often result from mechanisms like drug-drug and drug-disease interactions, reduced medication adherence, and complex medication regimens that may exceed a patient's capacity to manage [2,4,5]. The WHO has recognized polypharmacy as a major global health challenge, designating it as a critical area for drug safety intervention [6]. Consequently, various policies, including drug reduction protocols and incentivization schemes, have been implemented globally to mitigate its risks [7-10]. However, the impact of successful drug reduction on health outcomes remains uncertain, and while intervention trials are encouraged [2,11,12], their practical implementation is often challenging.

Geographical variability in polypharmacy prevalence has been highlighted by several studies. An ecological study in Italy, for instance, found regional variations in the prevalence of drug use across six categories [13], while data from a Swedish prescription database echoed these findings of regional disparity in polypharmacy [14]. Two observational studies applying Moran's I statistics at the municipal level reported spatial autocorrelation in polypharmacy [15,16], suggesting regional clustering of this issue. These studies have not fully elucidated the causes behind regional differences in polypharmacy; however, factors such as medication adherence [17] and socioeconomic disparities may offer some explanation [18]. Thus, spatial analysis provides added value by revealing geographic clustering and linking polypharmacy patterns to regional demographic and socioeconomic characteristics, which can inform targeted policy interventions.

Since medical resource allocation is organized by each local health unit (LHU), understanding regional characteristics is essential. Ecological studies play a critical role in visualizing regional health risks and offer insights into optimizing resource distribution for targeted interventions. Previous observational studies have primarily been conducted at the municipal level due to sample size constraints [14-18]. However, municipalities vary considerably in size and population, which may bias comparisons. The LHUs, defined as secondary medical care areas, are superior units of analysis because they align with healthcare delivery systems and population needs, thereby providing a more consistent framework for evaluating regional health issues. Moreover, most prior studies have been restricted to specific regions, raising concerns about generalizability.

The primary objective of this study was to analyze the spatial distribution of polypharmacy across Japan and to identify demographic, socioeconomic, and healthcare-related regional characteristics associated with higher polypharmacy rates. To address this, we conducted an ecological study using comprehensive open-access data from Japan, a country with a rapidly aging population. By examining spatial clustering and regional determinants, this study aims to generate evidence that supports geographically tailored interventions and informs healthcare policy.

This work was previously posted as a preprint on the Authoria server on November 19, 2024.

## Materials and methods

Study design

The Japanese Medical Law regulates the number of hospital beds and clinics within each of the 335 LHUs. Known as 'Niji Iryouken,' they differ in classification from local public administrative divisions. Based on these LHUs, we conducted an ecological study to assess the current status of polypharmacy in Japan. Both public and private hospitals are included under the universal health coverage system, and their data are reflected in the National Database (NDB). The datasets of our study do not include personal information, so anonymization was not necessary. Ethical review was waived as only open data without personal information was used. The study's findings have been reported in accordance with the Strengthening the Reporting of Observational studies in Epidemiology (STROBE) guidelines.

Data collection

Three open databases were utilized in this study. The first database was the NDB open data, a repository of health checkup and medical receipt information gathered by insurers and compiled by the Ministry of Health, Labour, and Welfare (https://www.mhlw.go.jp/index.html). As Japan has achieved universal health coverage, this nationwide dataset enables the aggregation of all medical care provided across the country. Data were aggregated by LHU, the most detailed classification available, representing regions where hospital and clinic bed distribution is regulated by the Japanese government. The second database was e-Stat, managed by the Statistics Bureau of the Ministry of Internal Affairs and Communications, which consolidates over 700 datasets from various government ministries and agencies, allowing comprehensive data searches and outputs (https://www.e-stat.go.jp/). The third was Geographic Information System (GIS) data provided by Environmental Systems Research Institute (ESRI) Japan Co. (https://www.esrij.com/). The GIS is a framework for gathering, managing, and analyzing spatial and geographic data. It enables the visualization of spatial distributions and relationships by linking geospatial coordinates with regional attributes. We used NDB Open Data from April 2022 to March 2023 to aggregate outpatient prescription counts and the results of specific health checkups conducted for Japanese citizens aged 40 and older, including body mass index, blood pressure, and blood test data. Using e-Stat, we collected demographic data (population size, age group distribution, number of physicians, and pharmacists), geographic data (area, population density, habitable area density, and number of hospitals and clinics), and socioeconomic data (highest educational level, employment status, and taxable annual income) from 2020 government statistics. These datasets, which do not overlap at the patient level, were integrated with ESRI’s 2015 geospatial data. All data were aggregated by LHU, with the highest educational attainment and employment status expressed as population proportions, while the number of physicians, pharmacists, hospitals, and clinics was standardized per 100,000 population.

Prescription data were further stratified by age in five-year increments to calculate the standardized polypharmacy count. In the NDB Open Data, polypharmacy prescriptions are defined as cases where seven or more medications are prescribed simultaneously. This threshold reflects the Japanese reimbursement policy, under which prescriptions with ≥7 drugs are reimbursed at a reduced rate compared to standard prescriptions as a measure to discourage unnecessary polypharmacy [7,19]. Notably, this cutoff lies between the definitions most frequently cited in the literature, where polypharmacy is often defined as five or more medications and 'megapharmacy' as 10 or more [1]. Based on this reimbursement criterion, we identified the number of polypharmacy prescriptions and calculated the standardized prescription ratio (SPR) using the following formula:

$SPR = \frac{Area Polypharmacy}{\sum_{AgeGrp=1}^{20}(AreaPopulation_{AgeGrp} * \frac{National Polypharmacy_{AgeGrp}}{National Population_{AgeGrp}})}$ [20].

The SPR standardizes the prevalence of polypharmacy prescriptions, enabling comparisons across regions. Since polypharmacy is generally influenced by age distribution, we estimated the expected number of polypharmacy prescriptions using five-year age-stratified population data for each region and divided the observed number by this predicted value. In other words, SPR represents the ratio of observed to expected polypharmacy prescriptions after adjusting for age distribution, allowing fair comparison across regions. To ensure the accuracy of this estimation, we evaluated the predictive performance of the expected polypharmacy values using the coefficient of determination (R²). We presented the representative values for each dataset according to the quartiles of SPR, using the mean and standard deviation.

Statistics

The research was carried out in two stages. First, a spatial epidemiological study was conducted to assess whether polypharmacy exhibited regional clustering patterns within each LHU. To account for spatial autocorrelation, we assessed it by calculating Moran’s I on the smoothed SPR [21]. The technical details of the model specification and simulation procedures are provided in Appendix A.

In the second stage, we employed a clustering method, a machine learning technique, to identify the characteristics of regions with prevalent polypharmacy prescriptions. We further visualized SPR across Japan using a color-coded map, allowing for visual confirmation of spatial patterns. To identify areas with a high SPR, we performed clustering using the k-means method. The optimal number of clusters was determined using the elbow method, and the clustering results were visualized using a plot based on principal component analysis (PCA) to assess the separation of clusters. Finally, based on this cluster, we visualized the features using a heatmap. The two-tailed significance level was set at p=0.05, and all statistical analyses were performed using R-4.3.1 (R Foundation for Statistical Computing, Vienna, AUT).

## Results

**Figure 1:**
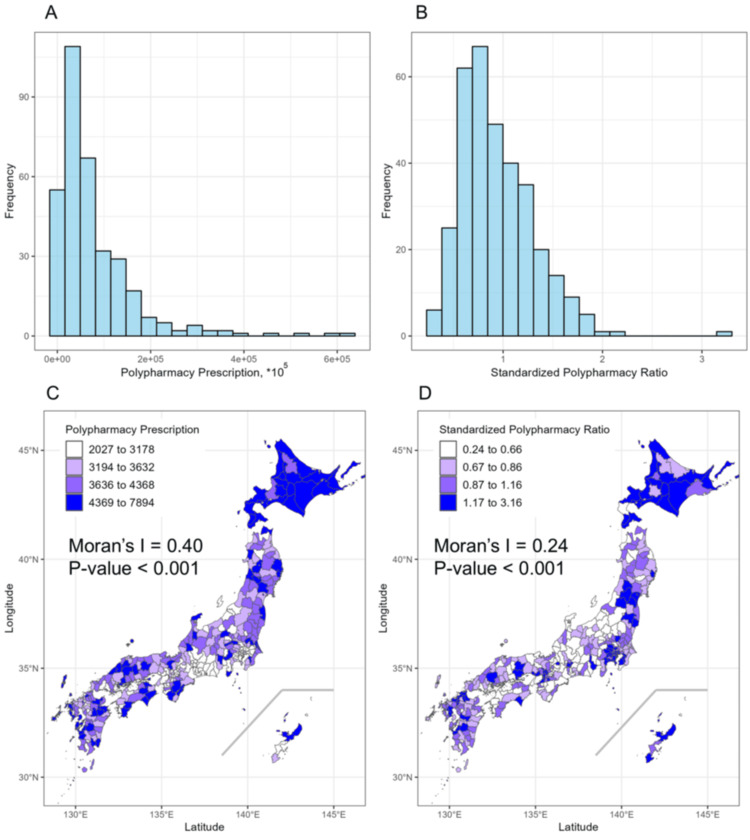
Distribution and spatial variation of polypharmacy prescriptions and SPR in Japan Panels A and B display the distributions of polypharmacy prescription counts and the SPR across regions. Panel A shows the skewed distribution of raw polypharmacy prescription counts, while panel B presents the distribution of SPR, which adjusts for regional age-group population and national prescription counts by age group, resulting in a closer approximation to a normal distribution. Panels C and D illustrate the geographic distribution of polypharmacy prescription counts and SPR in Japan. In panel C, regions with higher prescription counts are shown in darker shades, highlighting areas with a high polypharmacy burden. Panel D shows the SPR distribution, where darker shades indicate regions with an SPR above the national average, adjusted for age demographics. The base map used for spatial visualization was obtained from Natural Earth (https://www.naturalearthdata.com/downloads/10m-cultural-vectors/). SPR: Standardized polypharmacy ratio

Figure 1 presents the number of polypharmacy prescriptions across each LHU, a histogram of the SPR calculated based on age-group population and polypharmacy prescriptions by age group, the spatial distribution, and summary statistics. The median number of polypharmacy prescriptions per 100,000 people was 3,632, with an interquartile range of 3,194 to 4,368. The SPR had a median of 0.87, with an interquartile range of 0.67 to 1.17. The coefficient of determination (R²) for predicting the number of polypharmacy prescriptions was 0.98, indicating a high level of predictive accuracy.

To further investigate spatial patterns, we calculated the Moran statistic. Moran's statistics confirmed spatial autocorrelation, with a value of 0.43 for polypharmacy prescriptions (p<0.001) and 0.24 for SPR (p<0.001). Subsequently, the data were divided into quartiles based on SPR, with representative values for each quartile summarized in Table 1. Areas with higher SPR exhibited higher population density, more doctors and pharmacists, fewer individuals aged 60 and over, and a larger population aged 20 to 60. Higher SPR areas also had more university graduates, fewer people whose highest educational attainment was junior high or high school, a greater proportion of tertiary industry, lower primary and secondary industry, and higher income levels. No clear differences were observed between groups in specific health checkup results. All measured prescription types were more prevalent in high-SPR areas.

**Table 1:** Characteristics of LHUs stratified by interquartile range of the LHUs: Local health units; SPR: Standardized polypharmacy ratio; HbA1c, Hemoglobin A1c; sBP: Systolic blood pressure; HDL: High-density lipoprotein; LDL: Low-density lipoprotein; AST: Aspartate aminotransferase; ALT: Alanine aminotransferase; γ-GTP: Gamma-glutamyl transpeptidase; Hb: Hemoglobin; eGFR: Estimated glomerular filtration rate The values in parentheses represent standard deviations.

SPR	1st quartile (n=84)	2nd quartile (n=84)	3rd quartile (n=84)	4th quartile (n=83)
Geographical features				
Area, km^2^	1145 (850)	1160 (844)	914 (817)	1210 (1662)
Population density, persons/ha	7.3 (5.7)	8.6 (11.6)	16.7 (24.2)	38.6 (49.1)
Hospital, /10^5^ persons	8.2 (3.9)	8.9 (4.2)	8.7 (4.4)	7.9 (4.2)
Clinic, /10^5^ persons	79.5 (15.8)	79.3 (17.5)	80.6 (16.6)	79.6 (26.2)
Population, 10^3^ persons	205 (184)	255 (234)	369 (365)	681 (702)
-19 years, %	15.7 (2.1)	16.1 (1.7)	16.3 (2.1)	16.5 (2.2)
20-30 years, %	16.7 (2.6)	17.3 (2.5)	18 (3)	19.9 (3.8)
40-59 years, %	25.7 (1.7)	25.8 (1.9)	26.2 (2)	27.3 (2.2)
60-79 years, %	29.9 (3.3)	29.3 (3.1)	28.5 (3.8)	26.5 (4.2)
80- years, %	12.1 (2.8)	11.6 (2.4)	11 (2.7)	9.8 (2.4)
Physician, /10^5^ persons	201.2 (61.9)	223.4 (69)	247.2 (92.6)	259.1 (140.7)
Pharmacist, /10^5^ persons	187 (36.4)	201.7 (44.1)	216.6 (57.3)	246.7 (175.9)
Graduate, /10^5^ persons	82779 (2793)	82385 (2505)	81336 (3274)	80683 (3110)
Junior high school, %	18.7 (5.8)	17.7 (6.4)	16.5 (6.5)	14.1 (7.5)
High school, %	45 (4.4)	45.7 (4.9)	43.8 (6.9)	39 (9.4)
Professional school, %	13.2 (1.6)	13 (2.1)	13.1 (1.9)	13.1 (1.8)
College, %	15.3 (4.7)	15 (4.6)	16.6 (6.3)	19.5 (8.4)
Unknown, %	7.8 (3.6)	8.6 (4)	10 (5.6)	14.3 (7.6)
Working age, /10^5^ persons	50579 (2775)	49959 (2225)	48934 (3039)	47490 (3452)
Unemployed, %	3.5 (0.6)	3.7 (0.5)	3.9 (0.6)	4 (0.8)
Primary industry, %	7.5 (5.1)	8.4 (5.4)	6.5 (5.5)	5 (6.3)
Secondary industry, %	27 (7.1)	24 (6.2)	22.9 (6.2)	20.2 (5.7)
Third industry, %	59.9 (5.6)	61.8 (5.6)	64.4 (7)	68.1 (7.3)
Unknown, %	2.2 (1)	2.1 (1)	2.3 (1.1)	2.8 (1.1)
Taxable income, 10^3^ yen	2676 (359)	2696 (323)	2803 (442)	3242 (917)
Medical features				
BMI, kg/m^2^	23.4 (0.4)	23.5 (0.3)	23.5 (0.3)	23.6 (0.4)
HbA1c, %	5.7 (0.1)	5.7 (0.1)	5.7 (0.1)	5.7 (0.1)
sBP mmHg	127 (1.9)	126.8 (1.6)	126.4 (1.9)	125.8 (2.6)
Triglyceride, mg/dL	114.5 (5)	114.3 (4.9)	114.2 (4.5)	113 (5.4)
HDL, mg/dL	65.1 (1.2)	65.3 (1.3)	65.4 (1.2)	65.4 (1.2)
LDL, mg/dL	124.2 (1.9)	124.4 (1.9)	124.6 (1.8)	124.8 (1.6)
AST, U/L	23.8 (0.6)	23.9 (0.5)	23.9 (0.5)	23.9 (0.5)
ALT, U/L	23.6 (0.6)	24 (0.6)	24 (0.7)	24.3 (0.7)
γ-GTP, U/L	37.8 (2.3)	38.8 (2.1)	39 (2.4)	39.2 (2.2)
Hb, g/dL	14.1 (0.2)	14 (0.8)	14.1 (0.2)	14.1 (0.1)
eGFR, mL/min/1.73m²	74.7 (1.5)	75.1 (1.6)	75.1 (1.8)	75.6 (1.2)
Total prescriptions /10^5^ persons				
Prescription with over seven drugs	526969 (108155)	598566 (92893)	621448 (108334)	616064 (107573)
Prescription for infants	6708 (4054)	8662 (4807)	9419 (5653)	10574 (5093)
Opioid drugs	21995 (12225)	16893 (8548)	15062 (8102)	11976 (6529)
Anticancer drugs	1116 (1122)	1334 (1109)	1360 (1175)	1547 (1114)

**Figure 2:**
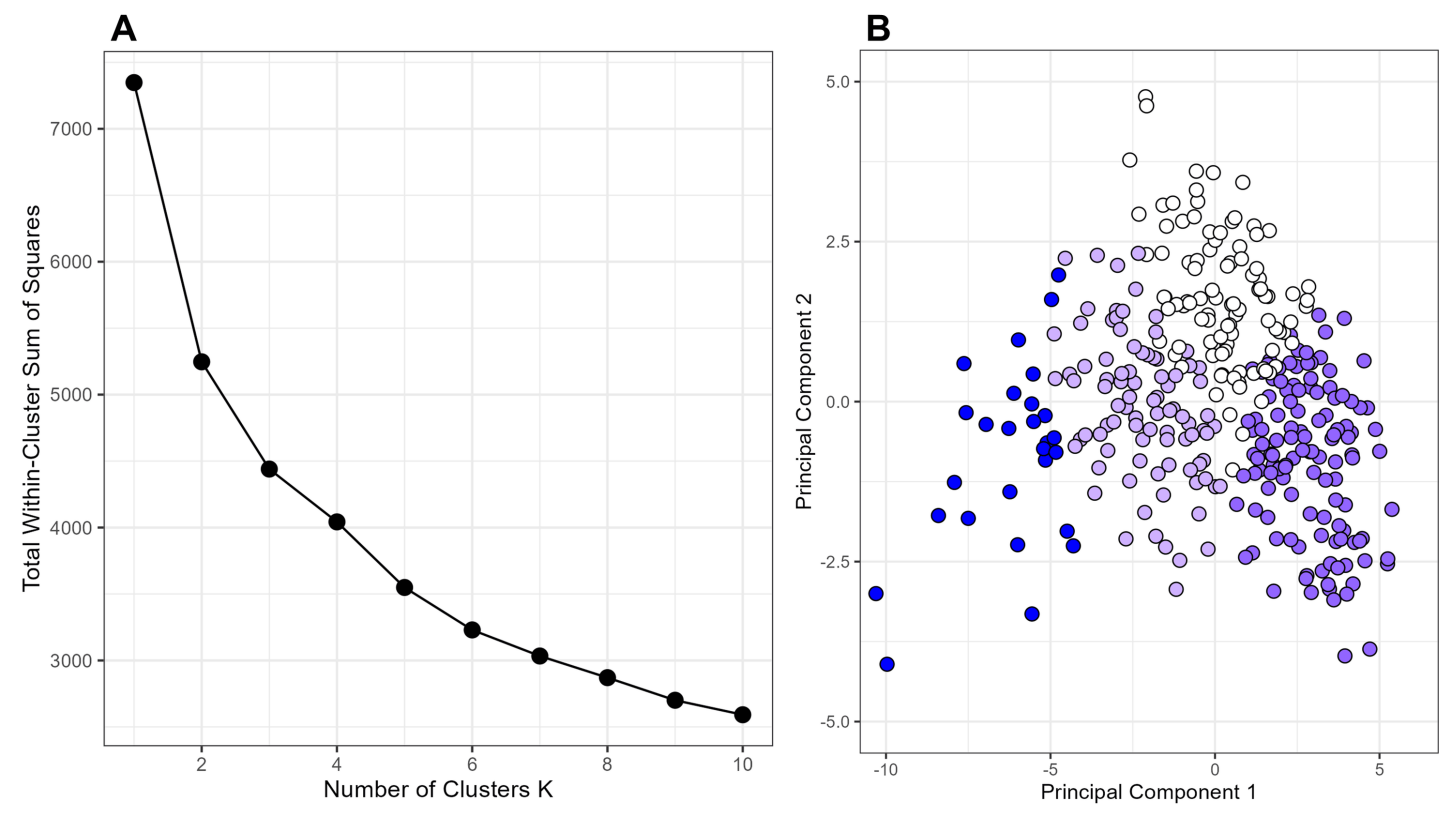
Determination and visualization of clustering structure in polypharmacy analysis This composite figure presents two key steps in the clustering analysis of polypharmacy-related features across LHUs in Japan. A: Elbow plot for determining the optimal number of clusters. The x-axis represents the number of clusters (K), and the y-axis indicates the total within-cluster sum of squares. As the number of clusters increases, the total within-cluster sum of squares decreases rapidly at first and then plateaus, indicating diminishing returns. The 'elbow' point, where the slope levels off (at K = 4), suggests an appropriate number of clusters for the dataset. B: The PCA of polypharmacy-related features. This scatter plot shows observations projected onto the first two principal components, capturing the most variance in the data. Each dot represents one LHU, and color shading reflects its cluster assignment. The PCA visualization helps illustrate the separation and structure of clusters derived from polypharmacy-related variables, supporting interpretation of regional patterns. LHU: Local health unit, PCA: Principal component analysis

**Figure 3:**
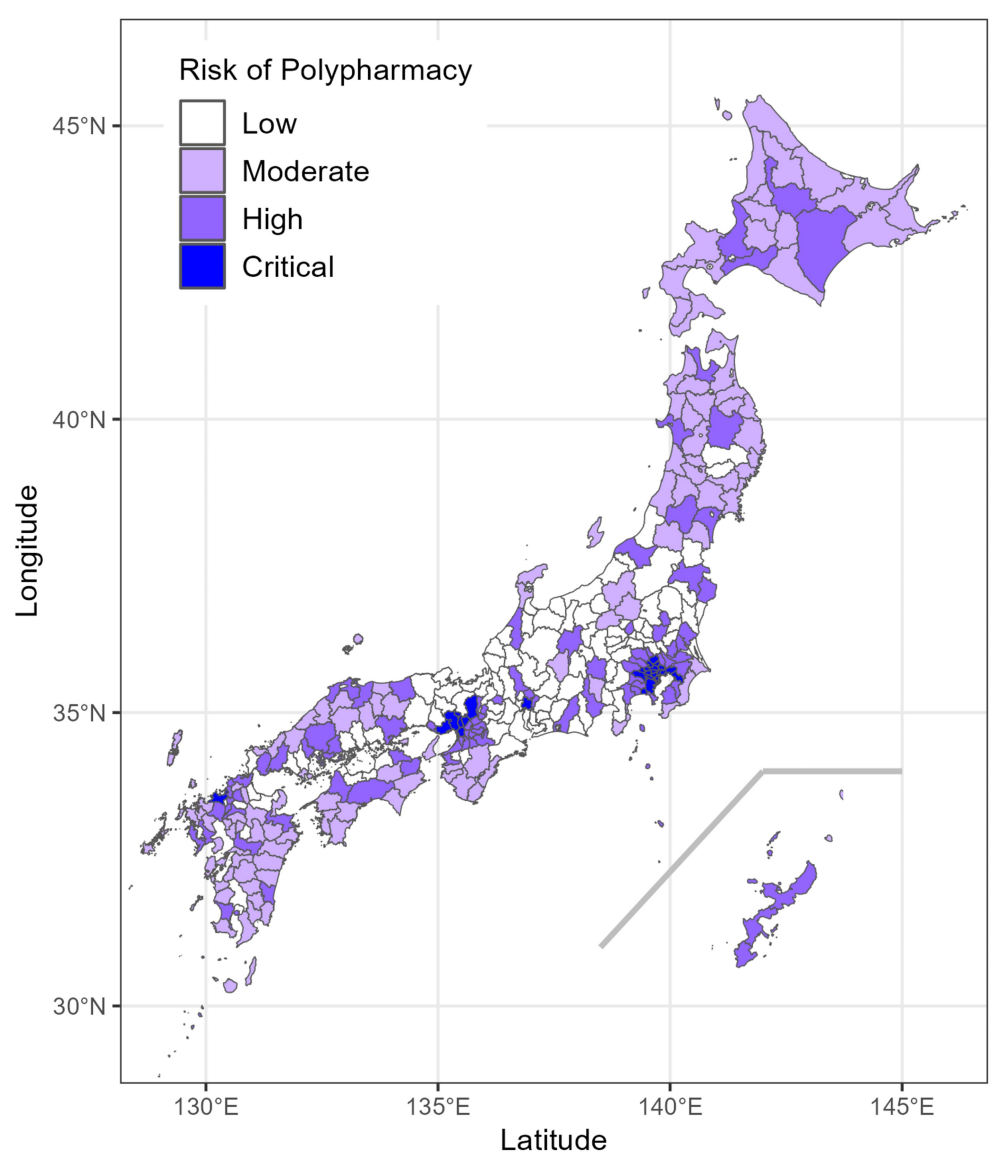
Spatial distribution of polypharmacy risk in Japan This map shows the spatial distribution of polypharmacy risk levels across Japan, categorized into four levels, namely low, moderate, high, and critical. Regions are color-coded with darker shades representing higher risk levels. The base map used for spatial visualization was obtained from Natural Earth (https://www.naturalearthdata.com/downloads/10m-cultural-vectors/).

Then, we performed clustering analysis focusing on variables that showed a potential association with SPR. Based on the elbow method (Figure 2A), we determined the optimal number of clusters to be four, categorizing them as critical, high, moderate, and low groups based on polypharmacy risk levels. The first two principal components accounted for a substantial proportion of the variance in the data, allowing for a clear visualization of clustering structures (Figure 2B). The mean (standard deviation) SPR values were as follows: critical, 1.37 (0.42); high, 1.11 (0.32); moderate, 0.90 (0.34); and low, 0.70 (0.17) (Table 2). The spatial distribution of these clusters, shown in Figure 3, closely resembles the spatial distribution of polypharmacy prescriptions and SPR presented in Figure 1.

**Table 2:** Characteristics of LHUs stratified by risk of polypharmacy LHUs: Local health units; SPR: Standardized polypharmacy ratio; HbA1c: Hemoglobin A1c; sBP: Systolic blood pressure; HDL: High-density lipoprotein; LDL: Low-density lipoprotein; AST: Aspartate aminotransferase; ALT: Alanine aminotransferase; γ-GTP: Gamma-glutamyl transpeptidase; Hb: Hemoglobin; eGFR: Estimated glomerular filtration rate. The values in parentheses represent standard deviations.

Risk of polypharmacy	Critical (n=28)	High (n=90)	Moderate (n=122)	Low (n=95)
SPR	1.37 (0.42)	1.11 (0.32)	0.90 (0.34)	0.70 (0.17)
1st quartile	0 (0.0)	5 (5.6)	30 (24.6)	49 (51.6)
2nd quartile	1 (3.6)	15 (16.7)	36 (29.5)	32 (33.7)
3rd quartile	6 (21.4)	32 (35.6)	33 (27.0)	13 (13.7)
4th quartile	21 (75.0)	38 (42.2)	23 (18.9)	1 (1.1)
Population density of habitable area, persons/m^2^	103.1 (43.8)	19.8 (15.7)	3.7 (2.1)	8.6 (4.7)
Population, %				
-19 years	16.6 (1.3)	17.5 (1.8)	14.6 (1.8)	16.7 (1.3)
20-30 years	24.2 (3.1)	19.7 (1.5)	15.1 (1.8)	18.2 (1.8)
40-59 years	29.8 (1.1)	27.2 (1.3)	24.4 (1.4)	26.5 (1.2)
60-79 years	21.7 (2.2)	26.5 (2.2)	32.1 (2.3)	28.1 (2.2)
80- years	7.7 (0.9)	9.2 (1.4)	13.8 (1.8)	10.5 (1.7)
Hospital, /10^5^ persons	4.5 (1.3)	7.2 (2.9)	11.4 (4.5)	7.0 (2.8)
Clinic, /10^5^ persons	90.7 (39.6)	79.3 (15.3)	81.6 (18.9)	74.7 (11.8)
Physician, /10^5^ persons	313 (209)	286 (93)	200 (55)	200 (49)
Pharmacist, /10^5^ persons	336 (278)	240 (53)	178 (32)	195 (36)
Highest educational attainment, %				
Junior high school	6.8 (2.1)	11.95 (2.9)	23.3 (5.2)	15.9 (3.3)
High school	27.0 (5.0)	40.2 (4.9)	48.3 (3.6)	45.0 (3.2)
Professional school	13.9 (1.6)	14.3 (1.4)	11.5 (1.3)	13.8 (1.5)
College	30.1 (4.6)	20.3 (4.4)	11.0 (2.1)	16.3 (2.9)
Employment types among the working, age %				
Unemployed	3.8 (0.5)	3.9 (0.6)	3.8 (0.8)	3.5 (0.5)
Primary industry	0.5 (0.3)	3.2 (2.4)	12.2 (5.2)	5.4 (3.5)
Secondary industry	16.4 (3.2)	21.5 (4.7)	21.4 (5.3)	30.3 (5.4)
Third industry	75.8 (3.4)	68.4 (4.4)	61.1 (5.2)	58.4 (4.5)
Taxable income, 10^3^ yen	4185 (1047)	3052 (274)	2458 (240)	2779 (252)

**Figure 4:**
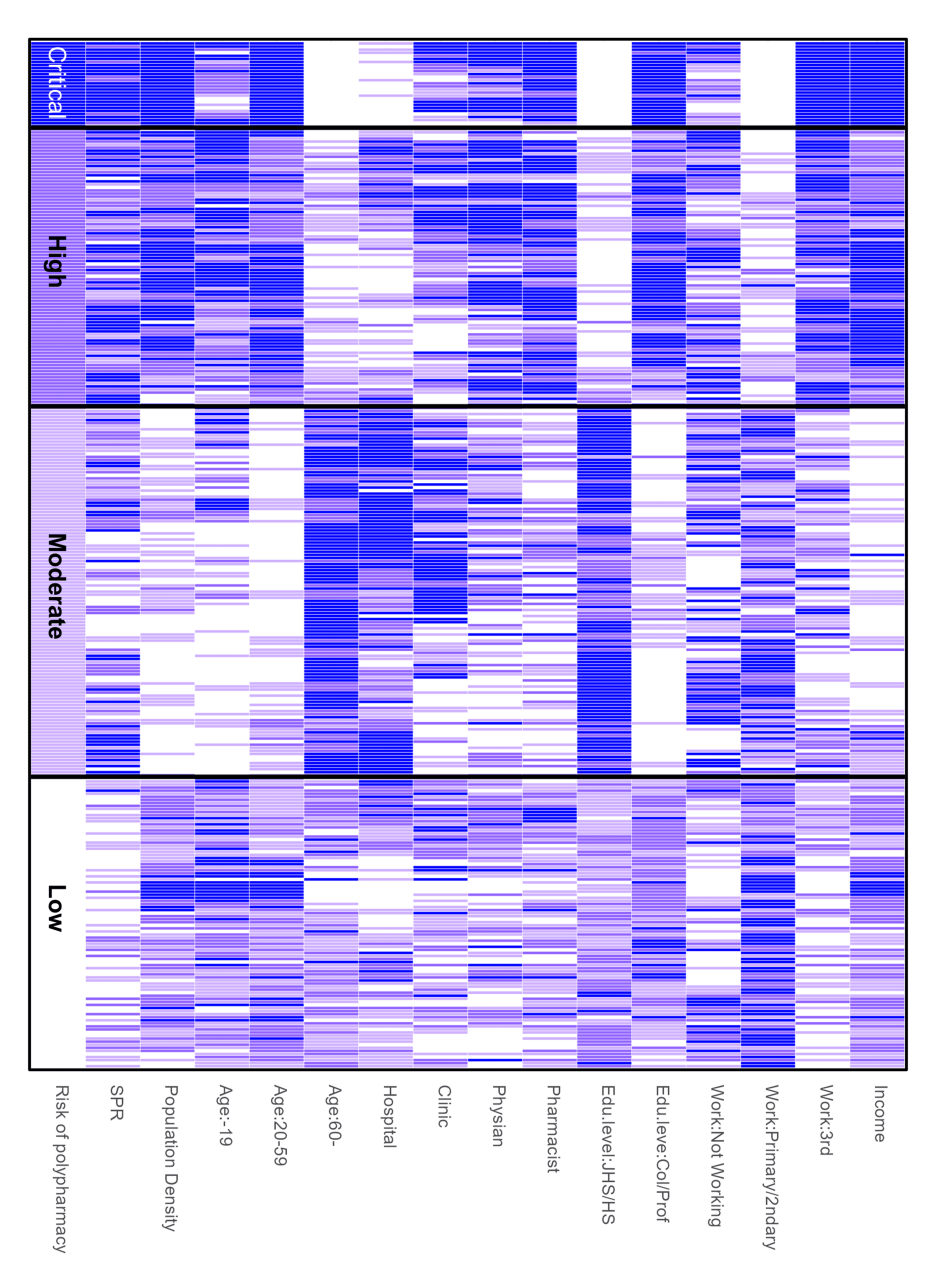
Heatmap of sociodemographic and healthcare characteristics by polypharmacy risk level This heatmap displays the distribution of various sociodemographic and healthcare characteristics across regions categorized by polypharmacy risk levels (low, moderate, high, and critical). Each row represents a region classified by its polypharmacy risk, while each column corresponds to a specific characteristic, including population density, age distribution, healthcare facility counts (hospitals, clinics), healthcare professionals (physicians, pharmacists), education levels, employment sectors, and income. Darker shades indicate higher values for each characteristic. The heatmap reveals distinct patterns across risk levels, illustrating the relationship between polypharmacy risk and regional sociodemographic and healthcare factors. SPR: Standardized polypharmacy ratio; Edu: Education; JHS: Junior high school; HS: High school; Prof: Professional; Col: College

Finally, we visualized the characteristics of LHUs by cluster in a heat map (Figure 4). The SPR increased with polypharmacy risk level. Population density and age distribution were positively associated with risk, although this relationship reversed in the moderate and low groups. The critical group consisted primarily of large cities with extremely high population density. In the critical and high groups, there was a predominance of individuals aged 20 to 60 and fewer people aged 60 and over, while this pattern was reversed in the moderate and low groups. The moderate group had many hospitals and clinics but fewer physicians and pharmacists. Educational attainment was highest in the critical and high groups, with the lowest rates in the moderate group. The proportion of workers in the tertiary industry increased with risk, whereas the proportion in the secondary industry decreased. Income was highest in the critical group, followed by the high, low, and moderate groups.

## Discussion

This study is an ecological investigation that clarified the current state of polypharmacy in Japan, a country with the highest proportion of elderly individuals worldwide. Using more comprehensive data than previous reports, we confirmed a spatial spread of polypharmacy and employed machine learning methods to identify its occurrence in areas of high population density. In addition, we demonstrated that polypharmacy prevalence was associated not only with age structure but also with socioeconomic conditions and healthcare resource availability, highlighting that both demographic and system-level factors contribute to its regional variability.

Geographic-level data frequently exhibit spatial correlation. Moran’s I statistic is a method for assessing the presence of spatial autocorrelation: a high correlation coefficient indicates similar distributions among neighboring regions, a coefficient near zero signifies a random distribution, and a negative coefficient reveals a 'marbled' pattern. Two prior studies have demonstrated spatial autocorrelation in polypharmacy. Feng et al. analyzed 37,570 Medicaid recipients in West Virginia and reported significant spatial autocorrelation (Moran’s I = 0.43, p < 0.01) [15]. Similarly, Franchi et al. analyzed data from 2 million individuals in the Lombardy region of Italy, using a drug management database to confirm spatial autocorrelation in polypharmacy prevalence at the municipal level (Moran's I = 0.36, p < 0.001) [16]. While these studies contributed valuable insights into spatial autocorrelation in polypharmacy, limitations were present in terms of spatial units (municipalities and groups rather than LHUs) and polypharmacy metrics (prevalence, patient counts). To address these limitations, we used LHUs as the analytical units and introduced the SPR to objectively depict regional variations in polypharmacy occurrence. The distribution of SPR more closely approximated a normal distribution than the raw polypharmacy data, although substantial variation persisted, highlighting clear geographical heterogeneity. The reduction of Moran’s I from 0.43 to 0.24 after standardization implies that the unstandardized variables’ spatial autocorrelation is influenced by age distribution and potentially overestimated. This finding, which remains statistically significant, underscores ongoing spatial propagation. Our results suggest the potential presence of exposure factors contributing to the observed spatial autocorrelation, even after appropriate standardization with high predictive accuracy (R² = 0.98).

Next, we conducted a clustering analysis based on features, including the SPR. Franchi et al. used statistical methods to infer causal relationships when identifying factors that reduce spatial autocorrelation, but concluded that the polypharmacy rate cannot be fully explained by health indicators alone [16]. Since causal inference through multivariable analysis may depend on the available explanatory variables, this study focused on descriptive statistics rather than causal inference, adopting a novel approach using machine learning. As a result of the clustering, we identified a group called 'critical,' characterized by a particularly high SPR, high population density, a greater proportion of young adults, a higher percentage of individuals with a college or technical school education, and a higher proportion of employment in the tertiary sector. This cluster includes major metropolitan areas. These findings are consistent with previous studies based on individual patient data, such as research in China reporting higher polypharmacy rates in urban areas [22,23]. Furthermore, it has been reported that mental disorders (e.g., mood and anxiety disorders) are more prevalent in urban than in rural areas, with poorer physical and functional health statuses [24,25]. It has also been reported that the risk of polypharmacy increases with visits to multiple healthcare facilities [26], which may explain our finding that the critical cluster had a high number of hospitals and clinics. These factors likely contribute to the prevalence of polypharmacy in urban areas. In contrast, studies in the U.S. targeting emergency department patients reported that rural residency is a risk factor for polypharmacy [27,28]. Findings on socioeconomic status (SES) are inconsistent; some studies associate high SES with polypharmacy, while others link it to low SES [29,30]. Such discrepancies highlight that results from individual-level studies are not uniform and may differ depending on regional and demographic contexts. In the context of policy decision-making, however, findings derived from ecological approaches using regional data are crucial. These findings are limited to proposing regional disparities and related hypotheses, and causal inferences cannot be drawn.

This study revealed marked regional disparities in the distribution of polypharmacy. Some previous policies addressing polypharmacy have targeted specific regions rather than the entire country, with some evolving into national policies [7-10]. Our study identified high-risk regions, indicating that targeting these regions for intervention may enhance the efficiency of interventions. The findings of this study underscore the significance of geographic variability in polypharmacy risk, suggesting that policymakers could increase intervention efficiency by targeting high-risk regions. In the Japanese context, this could include prioritizing reimbursement reforms that incentivize deprescribing, implementing region-specific audit and feedback programs for prescribers, and enhancing community-based initiatives such as pharmacist-led medication reviews. Moreover, the effects of such interventions may propagate within the region, resulting in lasting impacts.

The strengths of this study include its robust design, specifically the focus on LHUs and the introduction of the SPR, as well as its high coverage rate and large sample size. This study is the third to confirm spatial autocorrelation in polypharmacy [15,16], but previous research has only covered limited areas [13-17]. By analyzing data across all of Japan and leveraging the country's universal health insurance system, we were able to reflect the current healthcare landscape for the entire population accurately. Although the external validity of this study remains uncertain, the findings are noteworthy given that Japan has the highest proportion of elderly individuals worldwide and that the aging population is rapidly increasing in developed countries. Additionally, the availability of extensive government statistics in Japan enabled us to incorporate substantial regional data for clustering analysis.

Limitations

First, there are restrictions on data availability. This study defined polypharmacy as seven or more concurrent medications, a threshold that lies between conventional polypharmacy and 'megapharmacy' [1]. This definition is required in Japan due to the reimbursement policy [7,19], and alternative thresholds could not be applied. Furthermore, individual-level prescription data were not available, preventing calculation of patient-level polypharmacy prevalence, and important factors such as comorbidities, clinical practice patterns, and patient adherence could not be assessed, which may have influenced the observed associations. In addition, prescribing behaviors themselves may have been shaped by reimbursement structures, potentially biasing regional polypharmacy rates independent of actual clinical need.

Second, as with many ecological studies, causal inferences are challenging to establish. In particular, the reasons for increased polypharmacy in densely populated areas remain unclear. Several hypotheses may explain this, including higher levels of information literacy among patients and physicians, lifestyle factors, and social proximity. Although factors such as employment, income, and education level may partially account for these differences, deeper insights cannot be achieved with the current data. Moreover, whether interventions targeting regional characteristics such as socioeconomic status, mental health, or urban environments could resolve these disparities remains uncertain; causal relationships can only be clarified through longitudinal designs, such as pre-post policy evaluations or randomized controlled trials.

## Conclusions

This study provides a comprehensive analysis of polypharmacy across Japan, focusing on its spatial distribution and the demographic, socioeconomic, and healthcare factors associated with its prevalence. Our findings highlight that polypharmacy is not only prevalent in densely populated urban areas but also associated with factors such as medical resource availability and local population characteristics. Prioritizing high-SPR urban areas for intervention may be particularly cost-effective, given their concentration of high-risk populations and the potential for broad population-level benefits. Furthermore, the distribution of healthcare professionals, including pharmacists and physicians, plays a crucial role in addressing regional disparities in polypharmacy. Targeting high-risk regions with localized interventions may improve the effectiveness of polypharmacy management and potentially reduce adverse health outcomes associated with medication overload. Our results also underscore the importance of an evidence-based approach to policymaking in addressing polypharmacy at both regional and national levels.

## Appendices

Appendix A

Listed below are the supplementary methods with model specification and simulation procedures.

*Bayesian Spatial Modeling* To account for differences in municipality sizes and spatial dependence, we smoothed SPR values using a hierarchical Bayesian model. The neighborhood structure was incorporated by defining adjacency between LHUs, and Leroux’s conditional autoregressive (CAR) prior was applied to capture spatial correlation.

*Markov Chain Monte Carlo (MCMC) Procedures* The MCMC simulations were implemented with 120,000 iterations and a burn-in of 20,000. Convergence was assessed using trace plots and the Gelman-Rubin statistic. Posterior means were used as point estimates of smoothed SPR.

*Model Validation* The predictive performance of expected polypharmacy counts was evaluated using the coefficient of determination (R²), ensuring reliability of the standardized prescription ratios.
